# Prediction of T-cell epitopes of hepatitis C virus genotype 5a

**DOI:** 10.1186/1743-422X-11-187

**Published:** 2014-11-08

**Authors:** Maemu P Gededzha, M Jeffrey Mphahlele, Selokela G Selabe

**Affiliations:** HIV and Hepatitis Research Unit, Department of Virology, University of Limpopo, Medunsa Campus/National Health Laboratory Service, Pretoria, South Africa

**Keywords:** Hepatitis C virus, Genotype 5a, *In-silico*, T-cell epitopes, Vaccines

## Abstract

**Background:**

Hepatitis C virus (HCV) is a public health problem with almost 185 million people estimated to be infected worldwide and is one of the leading causes of hepatocellular carcinoma. Currently, there is no vaccine for HCV infection and the current treatment does not clear the infection in all patients. Because of the high diversity of HCV, protective vaccines will have to overcome significant viral antigenic diversities. The objective of this study was to predict T-cell epitopes from HCV genotype 5a sequences.

**Methods:**

HCV near full-length protein sequences were analyzed to predict T-cell epitopes that bind human leukocyte antigen (HLA) class I and HLA class II in HCV genotype 5a using Propred I and Propred, respectively. The Antigenicity score of all the predicted epitopes were analysed using VaxiJen v2.0. All antigenic predicted epitopes were analysed for conservation using the IEDB database in comparison with 406, 221, 98, 33, 45, 45 randomly selected sequences from each of the HCV genotypes 1a, 1b, 2, 3, 4 and 6 respectively, downloaded from the GenBank. For epitope prediction binding to common HLA alleles found in South Africa, the IEDB epitope analysis tool was used.

**Results:**

A total of 24 and 77 antigenic epitopes that bind HLA class I and HLA class II respectively were predicted. The highest number of HLA class I binding epitopes were predicted within the NS3 (63%), followed by NS5B (21%). For the HLA class II, the highest number of epitopes were predicted in the NS3 (30%) followed by the NS4B (23%) proteins. For conservation analysis, 8 and 31 predicted epitopes were conserved in different genotypes for HLA class I and HLA class II alleles respectively. Several epitopes bind with high affinity for both HLA class I alleles and HLA class II common in South Africa.

**Conclusion:**

The predicted conserved T-cell epitopes analysed in this study will contribute towards the future design of HCV vaccine candidates which will avoid variation in genotypes, which in turn will be capable of inducing broad HCV specific immune responses.

## Introduction

Hepatitis C virus (HCV) is estimated to infect 185 million people worldwide [1]. Chronic HCV infection leads to progressive liver disease, being one of the major causes of hepatocellular carcinoma and one of the most common indications for liver transplantation [2]. The World Health Organization (WHO) strongly recommends combination therapy with pegylated interferon and ribavirin for chronically infected patients who qualify for treatment [1]. Recently, two NS3 protease inhibitors (boceprevir and telaprevir) have been approved by the US Food and Drug Administration, and the WHO conditionally (until more evidence has accumulated) recommends that these drugs should be given in combination with pegylated interferon and ribavirin for the treatment of chronic HCV genotype 1 infections. Also, the WHO strongly recommends that sofosbuvir be given in combination with ribavirin alone in patients who cannot tolerate interferon and are chronically infected with genotypes 1, 2, 3 and 4 [1]. However, these therapies are still not affordable in most developing countries. As a result, the development of an effective HCV vaccine is undoubtedly the best solution for the ultimate control of HCV infections, and is a public health priority.

Prophylactic vaccines against viral infections are generally aimed at inducing a humoral (B-cell) immune response, while therapeutic vaccines preferably activate both humoral and cellular (T-cell) immune responses [3]. A successful HCV vaccine will need to stimulate both arms of the adaptive immune response, since while both cellular and humoral .immune responses occur in a naturally infected host, the current consensus is that a strong cellular response is vital for viral clearance and protection [4].

The development of an effective HCV vaccine requires an understanding of the host’s adaptive immune response to natural infection. As with other viral infections, viral antigens are presented to CD4+ and CD8+ T-cells via human leukocyte antigen (HLA) class II and class I molecules, respectively [5]. Different HLA class alleles have been found to be associated with HCV infection. For example, HLA-A*11, HLA-Cw*04 and HLA-B*53 have been associated with HCV persistence [6, 7], while HLA-B*27, HLA-A*11:01, HLA-B*57, HLA-Cw*01:02 and HLA-A*03 have been associated with spontaneous HCV clearance [8, 9]. Also, HLA-DRB1*11 and HLA-DQB1*03:01 have been associated with decreased disease severity of HCV infection globally, suggesting they may present HCV-derived epitopes more efficiently to CD4+ T-cells than others and thus capable of viral clearance [10]. During acute HCV infection, development and persistence of strong specific responses by CD8+ and CD4+ T-cells [11, 12] and neutralizing antibodies [13] are associated with viral clearance, with HCV-specific CD8+ and CD4+ T-cells usually being transient or absent in patients who develop persistent infections. The rate of chronic liver disease progression has been shown to be determined by the magnitude of HCV-specific CD4+ T-cell responses, since these cells are essential for both the cellular and humoral responses [14]. CD8+ T-cells are essential for long-term protection against chronic HCV [15], while CD4+ T-cells play a role in viral clearance [16].

HCV infection evades the host’s immune system by generating immune escape variants through alteration of the virus HLA-restricted epitopes to avoid being recognized by T-cells and neutralizing antibodies [14]. Thus effective HCV vaccines will need to target protective epitopes that display minimal cross-genotype amino acid variability as they will provide broad potency [17]. Peptides corresponding to protective epitopes are desirable vaccine candidates because they are easy to construct and produce, and they do not contain infectious materials [18]. The first step in the process of epitope-based vaccine design and development is the *in-silico* prediction of peptide binding affinities to HLA proteins [19].

Genotype 5 accounts for over 50% of HCV infections in South Africa [20], and is becoming more prevalent in Europe and North America [21]. It is the most conserved of HCV genotypes, being classified into only one subtype (5a) [22–24]. The growing prevalence of HCV genotype 5a in different parts of the world necessitates its molecular characterization in order to improve the formulations of vaccine candidates that are in development. Thus the aim of this study was to assess immunological determinants by predicting conserved epitopes in near-full length HCV genotype 5a sequences using a suite of online programmes to help in the designing of new vaccine candidates.

## Results

### Prediction of T-cell epitopes

For HLA class I, a total of 24 antigenic epitopes were predicted in the consensus near full-length of genotype 5a (Table 1). Epitope NS3^1325–1333^ covered 30 of the 47 HLA class I alleles that were analysed assuring high binding affinity to different alleles. For conservation analysis with other genotypes, 8 of 24 epitopes were 100% conserved for specific genotypes. Epitopes NS3^1332–1340^ and NS5B^2557–2565^ were highly conserved in all genotypes analysed, and epitope E2^684–692^ was conserved in all genotypes except for genotype 2, while 5 other epitopes were conserved in either 2 or 3 genotypes analysed. In addition epitope NS4B^1832–1840^ was conserved in genotype 1a, 1b, 2 and 4 epitope variants at anchor residues position 2 and 9 while E2^677–685^, NS3^1325–1333^ and NS3^1357–1365^ were conserved in at least 3 genotypes epitope variants each (Table 1). For HLA class II, 77 epitopes were predicted (Table 2). Epitope NS4B^1879–1887^ and NS4B^1880–1888^ covered 51 of 51 HLA class II alleles analysed. For conservation analysis with other genotypes, 31 of 71 epitopes were conserved for specific genotypes. Some epitopes were highly conserved in all genotypes (E2^507–515^, E2^509–517^, NS3^1253–1261^, NS3^1254–1262^, NS3^1327–1335^, NS3^1392–1400^, NS4B^1916–1924^ and NS4B^1919–1927^), while other epitopes were conserved in at least 1 to 4 of the genotypes analysed. Epitope NS4B^1886–1894^ was conserved at anchor residues position 1, 4, 6 and 9 in all 6 genotypes epitope variants while E2^692–700^, NS2^964–972^, NS3^1418–1426^ and NS4^1561–1569^ were conserved in at least 5 genotypes epitope variants each (Table 2). Epitopes NS3^1585–1593^, NS5A^2285–2293^ and NS5B^2889–2897^ were predicted to cover both HLA class I and HLA class II alleles. The highest number of HLA class I binding epitopes were predicted within the NS3 (63%), followed by NS5B (21%), and for the HLA class II, the highest number of epitopes were predicted in the NS3 (30%) followed by the NS4B (23%) proteins (Table 3).

**Table 1:** **HLA class I predicted epitopes of HCV genotype 5a and their antigenicity prediction score**, **number of allele and conservation** (**in percentages**) **in different genotypes**

Position	Epitope sequence	No of allele	Antigenecity score	Genotype 1a	Genotype 1b	Genotype 2	Genotype 3	Genotype 4	Genotype 6
**E2**									
651	**RCDLEDRDR**	9	2.7428						
677	**CSFTPTPAL**	27	0.8772	CSFT*T*LPAL (94)	CSFT*T*LPAL (92)		CSFTP*M*PAL (73)		
684	**ALSTGLIHL** ^#^ [25–27]	29	0.9005	**ALSTGLIHL** **(70)**	**ALSTGLIHL** **(99)**	**ALSTGLIHL** **(97)**	**ALSTGLIHL** **(97)**	**ALSTGLIHL** **(84)**	**ALSTGLIHL** **(91)**
**NS2**									
861	**VPPLQVRGG**	6	1.4312	VPPL*N*VRGG (85)					
**NS3**									
1032	**YAQQTRGVL**	26	0.7638	YAQQTRG*L*L (99)					
1033	**AQQTRGVLG**	5	0.7751	AQQTRG*L*LG (99)					
1128	**ADLYLVTRH**	5	1.0277	*S*DLYLVTRH (99)	*S*DLYLVTRH (99)	*V*DLYLVTR*N* (92)			
1325	**TILGIGTVL**	30	0.7555	*S*ILGIGTVL (93)		**TILGIGTVL** **(88)**	*S*ILGIGTVL (94)	**TILGIGTVL** **(82)**	
1332	**VLDQAETAG**	5	0.5004	**VLDQAETAG** **(96)**	**VLDQAETAG** **(98)**	**VLDQAETAG** **(96)**	**VLDQAETAG** **(100)**	**VLDQAETAG** **(93)**	**VLDQAETAG** **(71)**
1357	**TPHPNIEEV**	24	0.8423	*V*PHPNIEEV (89)	*V*PHPNIEEV (80)		*V*PH***S***NIEEV (94)		
1359	**HPNIEEVAL** ^#^ [28–31]	24	1.1654	**HPNIEEVAL** **(88)**	**HPNIEEVAL** **(76)**		H*S*NIEEVAL (94)		
1370	**EGEIPFYGR**	9	1.0560	*T*GEIPFYG*K* (97)					
1374	**PFYGRAIPL**	7	0.9479	PFYG*K*AIPL (99)	PFYG*K*AIP*I* (85)	PFYG*K*AIPL (82)			PFYG*K*AIPL (73)
1642	**TKYIMACMS**	6	0.7008	PFYG*K*AIPL (99)	**TKYIMACMS** **(70)**		**TKYIMACMS** **(70)**	**TKYIMACMS** **(93)**	TKYIM*T*CMS (87)
**NS4B**									
1712	**SASLPYMDE**	5	0.5488						
1819	**QIAPPTAAT**	5	0.5293	Q*L*A*A*P*G*AAT (99)	Q*L*APP*S*AA*S* (94)	QIAPP*AG*AT (91)		QIA*T*PTA*S*T (89)	
1832	**SGMAGAAVG**	5	0.8750	*A*G*L*AGAA*I*G (81)	*A*G*I*AGAAVG (93)	SG*LV*GAAVG (98)	SG*L*AGAA*I*G (76)		
1848	**LIDILAGYG**	5	0.8431	L*V*DILAGYG (94)	L*V*DILAGYG (95)		L*L*DILAGYG (79)	L*V*DILAGYG (84)	
1854	**GYGAGVAGA**	9	0.5562	**GYGAGVAGA** **(99)**	**GYGAGVAGA** **(99)**		GYGAGV*S*GA (94)	**GYGAGVAGA** **(91)**	GYGAGV*S*GA (84)
**NS5B**									
2522	**GYGAKEVRS**	7	1.3540	GYGAK*D*VR*C* (95)	GYGAK*D*VR*N* (84)	G*F*GAKEVRS (93)	GY*S*AK*D*VRS (79)		
2557	**TIMAKNEVF** ^#^ [32]	12	0.6303	**TIMAKNEVF** **(99)**	**TIMAKNEVF** **(80)**	**TIMAKNEVF** **(83)**	**TIMAKNEVF** **(100)**	**TIMAKNEVF** **(84)**	**TIMAKNEVF** **(93)**
2568	**EPSKGGKKP**	6	1.4218	*Q*P*E*KGG*R*KP (93)					
2720	**LASCRAAKL**	28	0.5264						
2886	**LHGLSAFSL**	11	0.6732	**LHGLSAFSL** **(99)**	**LHGLSAFSL** **(98)**			LHGLSAF*T*L(82)	LHG*MA*AFSL (98)

^#^-indicates that the epitope has been experimentally proven to be a true positive.

Bold- indicates percentage of epitope that is 100% conserved in more than 70% of the sequences analysed in each genotype.

Italic- indicates amino acid(s) variation in epitope in comparison to the predicted epitope.

**Table 2:** **HLA class II predicted epitopes of HCV genotype 5a and their antigenicity prediction score**, **number of allele and conservation** (**in percentages**) **in different genotypes**

Position	Epitope sequence	No of allele	Antigenecity score	Genotype 1a	Genotype 1b	Genotype 2	Genotype 3	Genotype 4	Genotype 6
E1									
320	**WDMMMNWSP**	10	1.3610	**WDMMMNWSP** **(99)**	**WDMMMNWSP** **(97)**		**WDMMMNWSP** **(97)**	**WDMMMNWSP** **(93)**	**WDMMMNWSP** **(93)**
**E2**									
507	**YCFTPSPVV**	6	1.2112	**YCFTPSPVV** **(93)**	**YCFTPSPVV** **(98)**	**YCFTPSPVV** **(88)**	**YCFTPSPVV (100)**	**YCFTPSPVV (91)**	**YCFTPSPVV (91)**
509	**FTPSPVVVG**	17	1.3553	**FTPSPVVVG (94)**	**FTPSPVVVG (98)**	**FTPSPVVVG (88)**	**FTPSPVVVG (100)**	**FTPSPVVVG (89)**	**FTPSPVVVG (73)**
665	**LLHTTTQWA**	5	0.6616	LL*LS*TTQW*Q* (100)			LLH*S*TT*EL*A (73)	LL*LS*TTQW*Q* (73)	
666	**LHTTTQWAI**	25	0.5863	L*L*TTTQW*Q*I (100)			LH*S*TT*EL*AI (73)		
692	**LHQNIVDTQ**	6	0.8564	LHQNIVD*V*Q (100)	LHQNIVD*V*Q (73)	LHQNIVD*V*Q (95)	LHQNIVD*V*Q (100)	LHQNIVD*V*Q (87)	
739	**LLVCQAEAA**	22	0.5506	LL*IS*QAEAA (100)	LL*IA*QAEAA (72)		L*MIS*QAEAA (73)		
**P7**									
803	**LPHRALALD**	7	0.6966	LP*Q*RA*Y*ALD (100)	LP*P*RA*Y*A*M*D (86)				
**NS2**									
860	**WVPPLQVRG**	7	1.1998	WVPPL*N*VRG (100)			WVPPL*LA*RG (82)		
861	**VPPLQVRGG**	25	1.4312	VPPL*N*VRGG (100)					
864	**LQVRGGRDA**	20	0.6368	L*N*VRGGRDA (100)	L*N*VRGGRDA (86)				
880	**FHPALGFEI**	19	1.4374	*V*HPAL*V*F*D*I (99)					
892	**LLGILGPLY**	14	0.5940	LL*AV*LGPL*W* (100)			L*IAV*LGPLY (82)		
895	**ILGPLYLLQ**	5	0.8264	*V*LGPL*WV*LQ (99)			*V*LGPLYL*I*Q (88)		
938	**LLHLGRLTG**	25	0.5554						
964	**LRDLAVATE**	9	1.0497	LRDLAVA*V*E (100)	LRDLAVA*V*E (93)	LRDLAVA*V*E (85)	L*K*DLAVATE (73)		LRDLAVA*V*E (93)
997	**LAGLPVSAR**	11	0.9120	*IN*GLPVSAR (100)			L*C*GLPVSAR (100)		
1025	**LLAPITAYA**	22	0.5253	**LLAPITAYA (99)**			**LLAPITAYA (79)**	**LLAPITAYA (89)**	
**NS3**									
1047	**LTGRDKNEA**	22	1.5633	LTGRDKN*QV* (100)	LTGRDKN*QV* (93)		LTGRDKN*VV* (90)		
1131	**YLVTRHADV** ^#^ [33–35]	15	0.7569	**YLVTRHADV (99)**	**YLVTRHADV (99)**	YLVTR*N*ADV (97)			
1152	**LLSPRPISY**	5	2.2999	**LLSPRPISY (99)**	LLSPRP*V*SY (73)	LLSPRP*L*S*T* (89)			
1153	**LSPRPISYL**	5	1.3109	**LSPRPISYL (99)**	LSPRP*V*SYL (73)	LSPRP*L*S*T*L (89)			
1253	**VLNPSVAAT**	14	1.2489	**VLNPSVAAT (100)**	**VLNPSVAAT (100)**	**VLNPSVAAT (97)**	**VLNPSVAAT (100)**	**VLNPSVAAT (96)**	**VLNPSVAAT (89)**
1254	**LNPSVAATL**	6	0.9169	**LNPSVAATL (100)**	**LNPSVAATL (100)**	**LNPSVAATL (98)**	**LNPSVAATL (100)**	**LNPSVAATL (95)**	**LNPSVAATL (89)**
1258	**VAATLGFGA**	11	1.2617	**VAATLGFGA (90)**	**VAATLGFGA (85)**	**VAATLGFGA(98)**			
1262	**LGFGAYMSK** ^#^ [36–38]	14	0.8784	**LGFGAYMSK (87)**	**LGFGAYMSK (82)**	**LGFGAYMSK(78)**			
1264	**FGAYMSKAY**	14	0.6561	FGAYMSKA*H* (100)	FGAYMSKA*H* (82)				
1327	**LGIGTVLDQ**	42	0.5607	**LGIGTVLDQ (97)**	**LGIGTVLDQ (88)**	**LGIGTVLDQ (98)**	**LGIGTVLDQ (100)**	**LGIGTVLDQ (93)**	**LGIGTVLDQ (96)**
1373	**IPFYGRAIP**	16	0.5679	IPFYG*K*AIP (100)	IPFYG*K*AIP (93)	IPFYG*K*AIP (81)			
1375	**FYGRAIPLA**	22	1.0280	FYG*K*AIPL*E* (100)	FYG*K*AIP*IE* (81)				FYG*K*AIPLE (71)
1392	**IFCHSKKKC**	22	1.5437	**IFCHSKKKC (93)**	**IFCHSKKKC (94)**	**IFCHSKKKC (95)**	**IFCHSKKKC (88)**	**IFCHSKKKC (80)**	**IFCHSKKKC (93)**
1415	**VAYYRGLDV**	21	0.8250	**VAYYRGLDV (98)**	**VAYYRGLDV (96)**	**VAYYRGLDV (92)**	**VAYYRGLDV (79)**	**VAYYRGLDV (91)**	VA*F*YRG*V*DV (89)
1418	**YRGLDVAVI**	51	1.0522	YRGLDV*S*VI (100)	YRGLDV*S*VI (97)	YRGLDV*S*VI (84)	YRGLDV*S*VI (85)	YRGLDV*S*VI (91)	YRG*V*DV*S*VI (67)
1482	**VSRSQRRGR**	12	2.0649	VSR*T*QRRGR (100)	**VSRSQRRGR (93)**	**VSRSQRRGR (96)**	**VSRSQRRGR (97)**	**VSRSQRRGR (98)**	**VSRSQRRGR (73)**
1561	**VFTGLTNID**	9	1.1689	VFTGLT*H*ID (100)	VFTGLT*H*ID (96)	VFTGLT*H*ID (98)	VFTGLT*H*ID (97)		VFTGLT*H*ID (89)
1583	**FPYLVAYQA**	24	0.6584		**FPYLVAYQA (85)**				FAYLVAYQA (78)
1585	**YLVAYQATV** ^#^ [25, 39]	17	0.5020	**YLVAYQATV (99)**	**YLVAYQATV (85)**	YL*T*AYQATV (70)		**YLVAYQATV (87)**	**YLVAYQATV (91)**
1586	**LVAYQATVC**	28	0.6052	**LVAYQATVC (99)**	**LVAYQATVC (85)**			**LVAYQATVC (82)**	**LVAYQATVC (91)**
1631	**VQNEITLTH**	35	1.1979	VQNE*V*TLTH (100)				VQNE*V*TLTH (80)	
1641	**ITKYIMACM**	8	0.6964	*V*TKYIM*T*CM (100)		*V*TKYI*AT*CM (94)		**ITKYIMACM (75)**	ITKYIM*T*CM (87)
1645	**IMACMSADL**	22	0.8406	IM*T*CMSADL (100)	**IMACMSADL (82)**	I*AT*CM*Q*ADL (99)	**IMACMSADL (70)**	**IMACMSADL (93)**	IM*T*CMSADL (87)
**NS4B**									
1733	**LGLIGTAGQ**	43	1.2450						
1765	**MWNFVSGIQ**	18	1.2300	MWNF*I*SGIQ (100)	MWNF*I*SGIQ (95)	MWNF*I*SGIQ (96)	**MWNFVSGIQ (100)**	MWNF*I*SGIQ (87)	
1766	**WNFVSGIQY**	22	0.6246	WNF*I*SGIQY (100)	WNF*I*SGIQY (95)	WNF*I*SGIQY (96)	**WNFVSGIQY (100)**	WNF*I*SGIQY (87)	**WNFVSGIQY (91)**
1791	**MSFTAAVTS**	8	0.7414	M*A*FTAAVTS (100)	M*A*FTA*SI*TS (88)	M*A*F*S*AA*L*TS (93)	M*A*FTA*S*VTS (92)	**MSFTAAVTS (93)**	
1812	**LGGWVASQI**	16	0.608	LGGWVA*A*Q*L* (100)	LGGWVA*A*Q*L* (95)		LGGWVA*THL* (88)	**LGGWVASQI (96)**	
1849	**IDILAGYGA**	9	0.7525	*V*DILAGYGA (100)	*V*DILAGYGA (97)		*L*DILAGYGA (79)	*V*DILAGYGA (84)	
1863	**LVAFKIMCG**	47	1.0160	LVAFKIM*S*G (100)		LVAFKIM*S*G (95)	LVAFKIM*G*G (100)	*V*V*T*FKIM*S*G (89)	LVAFKIM*S*G (84)
1879	**LVNLLPSIL**	51	0.7592	LVNLLP*A*IL (100)	LVNLLP*A*IL (90)		*M*VNLLP*A*IL (92)	LVNLLP*A*IL (73)	
1880	**VNLLPSILC**	51	0.9874	VNLLP*A*IL*S* (100)	VNLLP*A*IL*S* (92)		VNLLP*A*IL*S* (94)	VNLLP*A*IL*S* (89)	VNLLP*AL*L*S* (82)
1883	**LPSILCPGA**	6	0.7104	LP*A*IL*S*PGA (100)	LP*A*IL*S*PGA (99)	LPAIL*S*PGA (74)	LP*A*ILSPGA (100)	LP*A*IL*S*PGA (89)	LP*AL*L*S*PGA (84)
1886	**ILCPGALVV**	9	0.9365	IL*S*PGALVV (100)	IL*S*PGALVV (99)	IL*S*PGALVV (98)	IL*S*PGALVV (100)	IL*S*PGALVV (91)	*L*L*S*PGALVV (89)
1892	**LVVGVICAA**	30	0.7097	LVVGV*V*CAA (100)	LVVGV*V*CAA (98)	**LVVGVICAA (97)**	**LVVGVICAA (94)**	LVVGV*V*CAA (89)	LVVGV*V*CAA (93)
1893	**VVGVICAAV**	50	0.9530	VVGV*V*CAA*I* (100)	VVGV*V*CAA*I* (96)	VVGVICAA*I* (98)	VVGVICAA*I* (85)	VVGV*V*CAA*I* (91)	VVGV*V*CAA*I* (76)
1896	**VICAAVLRR**	35	0.9887	V*V*CAA*I*LRR (100)	V*V*CAA*I*LRR (95)	VICAA*I*LRR (97)	VICAA*I*LRR (85)	V*V*CAA*I*LRR (91)	V*V*CAAILRR (76)
1897	**ICAAVLRRH**	12	1.2316	*V*CAA*I*LRRH (100)	*V*CAA*I*LRRH (97)	ICAA*I*LRRH (97)	ICAA*I*LRRH (85)	*V*CAA*I*LRRH (89)	
1916	**MNRLIAFAS**	48	0.5291	**MNRLIAFAS (100)**	**MNRLIAFAS (99)**	**MNRLIAFAS (99)**	**MNRLIAFAS (100)**	**MNRLIAFAS (93)**	**MNRLIAFAS (96)**
1919	**LIAFASRGN**	24	2.0235	**LIAFASRGN (100)**	**LIAFASRGN (99)**	**LIAFASRGN (98)**	**LIAFASRGN (100)**	**LIAFASRGN (93)**	**LIAFASRGN (96)**
1922	**FASRGNHVS**	18	1.2938	**FASRGNHVS (100)**	**FASRGNHVS (98)**	FASRGNHV*A* **(97)**	**FASRGNHVS (94)**	**FASRGNHVS (82)**	**FASRGNHVS (93)**
**NS5A**									
1994	**WLQAKLLPQ**	33	1.1603	WL*K*AKL*M*PQ (100)	WLQ*S*KLLP*R* (84)				
2101	**YITGVTQDN**	6	1.1358	Y*V*TG*M*T*T*DN (100)					
2105	**VTQDNLKCP**	6	1.0178	*M*T*T*DNLKCP (100)					
2237	**MGGNITRVE**	20	1.2656	**MGGNITRVE (92)**	**MGGNITRVE (96)**		MG*S*NITRVE (91)		
2285	**LPVWARPGY**	9	0.6143	LP*I*WARP*D*Y (100)					LP*I*WARP*D*Y (78)
**NS5B**									
2422	**MSYSWTGAL**	9	0.5749	**MSYSWTGAL (76)**	MSY*T*WTGAL (94)	**MSYSWTGAL (97)**	**MSYSWTGAL (85)**	**MSYSWTGAL (91)**	
2451	**LRHHNLVYS**	40	1.2321	**LRHHNLVYS (89)**	LRHHN*M*VY*A* (86)		**LRHHNLVYS (97)**		
2523	**YGAKEVRSL**	24	1.1739	YGAK*D*VR*CH* (100)	YGAK*D*VR*N*L (85)	*F*GAKEVRSL (92)	Y*S*AK*D*VRSL (76)		
2559	**MAKNEVFAV**	18	0.8466	MAKNEVF*C*V (100)	MAKNEVF*C*V (81)	MAKNEVF*C*V (65)	MAKNEVF*C*V (85)		MAKNEVF*C*V (96)
2564	**VFAVEPSKG**	10	0.7925	VF*C*V*Q*P*E*KG (100)	VF*C*V*Q*P*E*KG (89)				
2637	**FSYDTRCFD**	11	15808	**FSYDTRCFD (99)**		**FSYDTRCFD (95)**	**FSYDTRCFD (100)**	**FSYDTRCFD (93)**	**FSYDTRCFD (87)**
2660	**YQSCDLQPE**	11	1.3802	YQ*C*CDL*D*P*Q* (100)	YQ*C*CDL*A*PE (94)				
2696	**YRRCRASGV**	41	1.2642	**YRRCRASGV (99)**	**YRRCRASGV (99)**	**YRRCRASGV (98)**	**YRRCRASGV (97)**		
2778	**YDLELVTSC**	6	1.0923	YDLEL*I*TSC (100)	YDLEL*I*TSC (96)	YDLEL*I*TSC (98)	YDLEL*I*TSC (91)		YDLEL*I*TSC (96)
2850	**FSVLQSQEQ**	21	0.8451		FS*I*L*LA*QEQ (84)			FS*I*LQSQE*A* (78)	
2871	**VYSVTPLDL**	29	2.0113	*C*YS*IE*PLDL (100)	*C*YS*IE*PLDL (75)		*T*YSVTPLDL (91)	*T*YS*I*TPLDL (87)	
2883	**IQRLHGLSA**	50	0.4322	**IQRLHGLSA (98)**	**IQRLHGLSA (72)**	IQ*Y*L*A*GLS*T* (93)	I*E*RLHGLSA (79)	**IQRLHGLSA (75)**	IQRLHG*MA*A (96)
2889	**LSAFSLHSY** ^#^ [40]	6	0.6007	**LSAFSLHSY (99)**	**LSAFSLHSY (97)**			LSAF*T*LH*G*Y (80)	*MA*AFSLH*G*Y (89)

^#^-indicates that the epitope has been experimentally proven to be a true positive.

Bold- indicates percentage of epitope that is 100% conserved in more than 70% of the sequences analysed in each genotype.

Italic- indicates amino acid(s) variation in epitope in comparison to the predicted epitope.

**Table 3:** **Distribution of genotype 5a HLA class I and II predicted epitopes in each of the HCV gene**

HLA class	No of predicted epitopes	Distribution of the predicted epitopes in each of the HCV gene ^a^									
		C	E1	E2	P7	NS2	NS3 ^b^	NS4A	NS4B	NS5A	NS5B
I	24	0 (0)	0 (0)	3 (12)	0 (0)	1 (4)	**15** **(63)**	0 (0)	0 (0)	0 (0)	5 (21)
II	77	0 (0)	1 (1)	6 (8)	1 (1)	10 (13)	**23** **(30)**	0 (0)	18 (23)	5 (7)	13 (7)

^a^Number of epitopes and their percentage in brackets.

^b^HCV gene with highest number of binding epitopes (in bold).

### Epitope binding affinity to common South African HLA class I and HLA class II alleles

Genotype 5 epitopes and their genotypic variants were analysed for their binding affinity to HLA class I and HLA class II alleles most common in South Africa, where genotype 5a is predominating. For HLA class I, 11 of the most common South African HLA-A alleles (HLA-A*01:01, HLA-A*02:01, HLA-A*30:01 and HLA-A*30:02), HLA-B (HLA-B*0702, HLA-B*08:01 and HLA-B*3501), and HLA-C (HLA-C*04:01, HLA-C*06:01, HLA-C*07:01 and HLA-C*07:02) were analysed. The limitation of Propred 1 is that it does not cover most of the main HLA class I alleles: HLA-A (HLA-A*01:01, HLA-A*30:01 and HLA-A*30:02), HLA-B (HLA-B*08:01) and HLA-C (HLA-C*04:01, HLA-C*06:01, HLA-C*07:01 and HLA-C*07:02) that are observed in South Africa. As a results the IEDB epitope analysis tool was used to predict epitopes of all the 11 most common HLA-A, HLA-B, HLA-C covering HLA class I alleles found in South Africa with the ANN prediction server. Thirteen antigenic epitopes with high binding affinity score of <50 IC_50_nM were predicted. Most epitopes bind with high affinity to single HLA class I alleles with exception of epitope NS5B^2889–2297^ LSAFSLHSY that bind with high affinity to 2 HLA-A alleles (HLA-A*01:01 and HLA-A*30:02). Four of the epitopes binding to HLA-B*35:01 followed by HLA-A*02:01 with 3 epitopes binding to it, however, none of the epitopes bind to HLA-C*04:01, HLA-C*06:01 and HLA-C*07:02 alleles. The NS3^1359–1367^ shows a level of promiscuity to HLA-A and HLA-B alleles. NS3^1359–1367^ HPNIEEVAL bind with intermediate affinity to HLA-B*07:02, while its genotype 2, 3 and 4 variant H*S*NIEEVAL bind with poor affinity and genotype 6 variant HPNI*T*E*T*AL bind with high affinity. For HLA-B*35:01, the NS3^1359–1367^ HPNIEEVAL and genotype 6 variant HPNI*T*E*T*AL bind with high affinity while genotype 2, 3 and 4 variant H*S*NIEEVAL bind with poor affinity. Three (E2^684–692^, NS3^1032–1040^ and NS3^1359–1367^) of the thirteen epitopes were predicted by both Propred 1 and IEDB analysis tool (Table 4).

**Table 4:** **Binding affinity scores of predicted epitopes and their variants to common HLA I allele types prevalent in South Africa**

Gene	Epitope sequence ^a^	Genotype of epitope	HLA I allele types ^b^										
			HLA-A				HLA-B			HLA-C			
			HLA-A*01:01	HLA-A*02:01	HLA-A*30:01	HLA-A*30:02	HLA-B*07:02	HLA-B*08:01	HLA-B*35:01	HLA-C*04:01	HLA-C*06:02	HLA-C*07:01	HLA-C*07:02
**E2**													
684	ALSTGLIHL^#^ [25–27]	1a, 1b, 3, 4,5a, 6	+	+++	+	+	+	+	+	+	+	+	+
	ALSTGL*L*HL	2	+	+++	+	+	+	+	+	+	+	+	+
**NS2**													
1025	LLAPITAYA	1a, 3, 4, 5a	+	+++	+	++	+	+	+	+	+	+	+
	LLAPITAY*T*	2	+	+++	+	++	+	+	+	+	+	+	+
**NS3**													
1032	YAQQTRGVL	5a	+	+	+	+	+++	+	+	+	+	+	+
	YAQQTRG*L*L	1a, 2	+	+	+	+	++	+	+	+	+	+	+
	Y*S*QQTRG*L*L	1b	+	+	+	+	+	+	+	+	+	+	+
	YAQQTRG*LV*	6	+	+	+	+	+	+	+	+	+	+	+
1242	AAYAAQGYK	1a, 1b, 4, 5a	+	+	+++	+	+	+	+	+	+	+	+
	AAYA*S*QGYK	2	+	+	+++	+	+	+	+	+	+	+	+
1264	FGAYMSKAY	5a	+	+	+	+	+	+	+++	+	+	+	+
	FGAYMSKA*H*	1a, 1b, 2	+	+	+	+	+	+	+	+	+	+	+
1359	HPNIEEVAL^#^ [28–31]	1a, 1b, 5a	+	+	+	+	++	+	+++	+	+	+	+
	H*S*NIEEVAL	2, 3, 4	+	+	+	+	+	+	+	+	+	+	+
	HPNI*T*E*T*AL	6	+	+	+	+	+++	+	+++	+	+	+	+
1367	LPSEGEIPF	5a	+	+	+	+	++		+++	+	+	+	+
	L*GH*EGEIPF	2	+	+	+	+	+	+	++	+	+	+	+
	L*G*SEGEIPF	3	+	+	+	+	+	+	++	+	+	+	+
	LP*TT*GEIPF	4, 6	+	+	+	+	++		+++	+	+	+	+
1585	YLVAYQATV^#^ [25, 39]	1a, 1b, 4, 5a, 6	+	+++	+	+	+	+	+	+	+	+	+
	YL*T*AYQATV	2, 3	+	+++	+	+	+	+	+	+	+	+	+
**NS5A**													
1927	NHVSPTHYV	1a, 1b, 3, 4,5a, 6	+	+	+	+	+	+	+	+	++	+++	+
	NHV*A*PTHYV	2	+	+	+	+	+	+	+	+	+	++	+
2285	LPVWARPGY	5a	+	+	+	+	+	+	+++	+	+	+	+
	LP*I*WARP*D*Y	1a, 3, 4, 6	+	+	+	+	+	+	+++	+	+	+	+
	LP*A*WARP*D*Y	2	+	+	+	+	+	+	+++	+	+	+	+
**NS5B**													
2696	YRRCRASGV	1a, 1b, 2, 3, 5a	+	+	+	+	+	++	+	+	++	+++	++
2763	MTRYSAPPG	1a, 1b, 2, 3, 4, 5a, 6	+	+	+++	+	+	+	+	+	+	+	+
2889	LSAFSLHSY^#^ [40]	1a, 1b, 5a	+++	+	+	+++	+	+	++	+	+	+	+
	LSAF*T*LHSY	3	+++	+	+	+++	+	+	++	+	+	+	+
	LSAF*T*LH*G*Y	4	+++	+	+	+++	+	+	+	+	+	+	+

^#^-indicates that the epitope has been experimentally proven to be a true positive.

^a^–italics indicates amino acid(s) variation in epitope in comparison to the predicted epitope.

^b^+++ indicates high binding affinity (<50 IC_50_nm), ++ indicates medium binding affinity (>50 IC_50_nm, <500 IC_50_nm), + indicates poor binding affinity (>500 IC_50_nm).

For HLA class II alleles, 4 most common HLA-DRB alleles (HLA-DRB1*03:01, HLA-DRB1*04:01, HLA-DRB1*11:01 and HLA-DRB1*15:01) were analysed. Nineteen antigenic epitopes with high binding affinity score of <50 IC_50_nM were predicted. The HLA-DRB1*11:01 has the highest number of binding epitopes (10) followed by HLA-DRB1*04:01 and HLA-DRB1*15:01 (7 each). Epitopes NS4B^1919–1927^ LIAFASRGN was predicted to be the most promiscuous epitope binding to HLA-DRB1*04:01, HLA-DRB1*11:01 and HLA-DRB1*15:01 with high affinity and HLA-DRB1*03:01 with intermediate affinity. This epitope is conserved in all genotypes. Epitopes E2^507-515^, NS4^B1774–1782^, NS4B^1919–1927^, and NS4B^1920–1928^ were highly conserved in all genotypes (Table 5).

**Table 5:** **Binding affinity scores of predicted epitopes and their variants to common HLA class II allele types prevalent in South Africa**

Position	Predicted epitopes	Genotype of epitope	HLA II- DRB1 allele types ^a^			
			HLA-DRB1*03:01	HLA-DRB1*04:01	HLA-DRB1*11:01	HLA-DRB1*15:01
**E2**						
507	YCFTPSPVV	1a, 1b, 2, 3, 4, 5a, 6	+	+++	+	+++
695	NIVDTQYLY	5a	+	+++	+	+++
**NS2**						
938	LLHLGRLTG	5a	+	++	+++	+
1025	LLAPITAYA	1a, 3, 4, 5a,	+	++	+++	++
**NS3**						
1129	DLYLVTRHA	1a, 1b, 5a	+	+	+++	+
1131	YLVTRHADV	1a, 1b, 5a	+	+	+++	+
1391	LIFCHSKKK	1b, 2, 3, 4, 5a, 6	+	+	+++	+
1417	YYRGLDVAV	5a	+	+++	++	+
1464	FSLDPTFTI	1a, 1b, 2, 5a	+	+++	+++	+
1535	TTVRLRAYL	3, 5a, 6	+	+	+	+++
1562	FTGLTNIDA	5a	+	+++	++	+
1666	VVAALAAYC	5a	+	+	+	+++
**NS4B**						
1774	YLAGLSTLP	1a, 1b, 2, 3, 4, 5a, 6	+	+++	+	+
1919	LIAFASRGN	1a, 1b, 2, 3, 4, 5a, 6	++	+++	+++	+++
1920	IAFASRGNH	1a, 1b, 2, 3, 4, 5a, 6	+	+	+++	+
**NS5A**						
1994	WLQAKLLPQ	5a	+	++	+++	+
2099	YHYITGVTQ	5a	+	++	+++	++
**NS5B**						
2450	LLRHHNLVY	1a, 3, 5a	+	++	++	+++
2579	LIVYPDLGV	2, 3, 4, 5a, 6	+	+	+	+++

^a^+++ indicates high binding affinity (<50 IC_50_nm), ++ indicates medium binding affinity (>50 IC_50_nm, <500 IC_50_nm), + indicates poor binding affinity (>500 IC_50_nm).

### Validation of epitopes

Seven of the predicted epitopes were previously confirmed experimentally by other studies as true positives in comparison with the epitopes analysed in the IEDB resource database. Majority of the epitopes predicted in this study have not been previously tested experimentally. The ‘true epitopes’ are highlighted by (^#^) in Tables 1, 2 and 4.

## Discussion

Several studies that have published HCV epitopes focused mainly on genotype 1 [41, 42], but most of these studies do not take into account the diversity in other genotypes that are common in developing countries like most African countries. In the present study, predicted antigenic epitopes of HCV genotype 5a proteins from South Africa were analysed followed by conservation with randomly selected genotypes 1–6 references from GenBank. Several studies have confirmed the importance of using immunoinformatics as good predictors for selecting HLA ligands, T-cell epitopes and immunogenicity [43]. As a result, several immunoinformatics methods have been developed to assist in the identification of HLA binding peptides [44, 45].

For this analysis, near full-length sequences covering all HCV proteins with the exclusion of the 3′end of the NS5B were included to maximize number of epitopes predicted. The use of the whole viral genome for developing epitope vaccines has a potential control over the immune response and eliminating the side effects [43], and it also increases the chance of detecting a virus at any developmental stage [46]. It has been shown that multiple epitopes from different parts of the HCV genome are important to produce a vaccine that can elicit strong humoral immune responses and multiple specific cellular immune responses [47]. A polyepitope-based strategy with multiple components combining core, E1, and E2 proteins; and conserved T-cell epitopes in the NS proteins has been suggested to be a good vaccine candidate for HCV [48].

High number of epitopes was predicted for HLA class II as compared to class I. The findings of this study are consistent with a study by Shehzadi et al. that predicted epitopes in genotype 3 from Pakistan. The study showed that majority of predicted epitopes were found in the NS3 protein for both HLA class I and HLA class II alleles and most of the epitopes were conserved among different genotypes [49]. Although the NS3 region is one of the conserved regions in HCV, variability in the nucleotide and amino acids has been reported by several studies in the same genotype and also in different genotypes [50, 51]. A recent study that analysed 1568 NS3-protease sequences from genotypes 1–6 reported that the protease amino acids sequence was moderately conserved and majority of the amino acids clustered in small regions. Of the 181 amino acids analysed 47% showed <1% variability among all HCV genotypes, and 17.1% amino acid positions showing >25.1% variability [51]. The NS3 is considered to be a good cellular target candidate for a therapeutic vaccine [52] since majority of the HCV viral epitopes recognized by CD8+ and CD4+ T-cells are located in the NS3 region [53–56]. The NS3 specific CD4+ and CD8+ T-cell responses were reported in patient responders to interferon therapy [57] and in spontaneous clearance of HCV [58].

Most of the predicted epitopes in the study sequence were found to be conserved across different HCV genotypes with a higher number of epitopes conserved at the anchor residues. The anchor residues are important for epitope high binding affinity to HLA [59]. Conserved epitopes might influence the immunogenic potential since mutations within the epitopes can increase the chance of immune escape [60]. For a vaccine to be effective globally the selected epitopes must cover HLAs of different populations and it must also be conserved among different genotypes. The high mutation rates of viral epitopes and HLA polymorphisms are some of the challenges that are associated with the development of peptide vaccines [61]. Successful epitope vaccine design requires a broad knowledge of HCV genotype diversity. This will help in the proper selection of conserved HCV-specific T-cell epitopes that will help in avoiding HCV immune evasion [62]. This study attempted to ensure maximal coverage of HLA polymorphism and different genotypes by analyzing conserved epitopes considering different HLA alleles.

Majority of the epitopes predicted from HCV proteins isolated from South African genotype 5a were good binders against HLA alleles that are found worldwide. HLA is both polygenic and polymorphic, and the pool of HLA molecules differs for every individual. Different HLA alleles bind peptides with a particular sequence pattern [63]. For an HLA allele to be covered by a set of epitopes, at least one of the epitopes should be capable of inducing an immune response when bound to the corresponding HLA molecule [46]. The epitopes predicted in this study bind to many HLA alleles including the ones common in South Africa and can be used for designing good vaccine candidates that will eventually work in genetically diverse populations. *In*-*vitro* and *in-silico* studies have showed that HLA alleles preferentially bind to conserved regions of viral proteins in human viruses [64].

Very few epitopes were found to be experimentally true positive, however this can be due to the fact that most of the previous studies focused on genotype 1. A limitation of the study was a lack of *in*-*vivo* and *in*-*vitro* studies to confirm the predicted immunogenic epitopes, which will be the focus of future studies. However *in*-*silico* studies still provide the basis for designing good vaccine candidates.

In conclusion, the results of this study demonstrated antigenic T-cell epitopes that are conserved among genotypes and good HLA binders derived from genotype 5a sequences that can be good candidates for vaccine development. Predicted epitopes analysed in this study will contribute to the future design of an efficient vaccine with the use of conserved epitopes to avoid variation in genotypes and as such, it will be able to induce broad HCV specific immune responses. Conserved epitopes among different genotypes will be experimentally tested in the future to determine their involvement in immune response.

## Methods

### Ethical statement

The study was approved by the Medunsa Research and Ethics Committee of the Faculty of Health Sciences at the University of Limpopo as project no MREC/p/142/2009:PG. The MREC is registered as an Independent Review Board with a reference no (IRB00005122).

### Prediction of T-cell epitopes

Genotype 5a full-length sequences available in the GenBank and 6 of the near full length sequences generated from a previous study conducted by our group [24] were aligned and consensus sequences created using BioEdit [65] for the prediction of T-cell epitopes. For HLA class I, prediction for binding alleles was performed using ProPred I (http://www.imtech.res.in/raghava/propred1/) at a 4% default threshold by keeping the proteosome and immunoproteosome filters on at 5% threshold. ProPred 1 predicts antigenic epitopes for 47 HLA class I alleles [44]. For HLA class II, prediction was performed using ProPred (http://www.imtech.res.in/raghava/propred/) at a 3% default threshold. ProPred predicts antigenic epitopes for 51 HLA class II alleles [45].

### Antigenicity of the epitopes

The Antigenicity score of all the predicted epitopes were analysed using VaxiJen v2.0 online antigen prediction (http://www.ddg-pharmfac.net/vaxijen/). Epitopes having antigenic score >0.5 were selected as antigenic. Vaxijen server performed well with 87% accuracy at a threshold of 0.5 antigenic score for viruses. VaxiJen v2.0 allows antigen classification based on the physicochemical properties of proteins without recourse to sequence alignment.

### Epitope conservation analysis

All predicted epitopes were analyzed for conservation using the IEDB database (http://tools.immuneepitope.org/tools/conservancy/iedb_input) at a threshold of 100% conservation in comparison with 406, 221, 98, 33, 45, 45 randomly selected sequences from each of the HCV genotypes 1a, 1b, 2, 3, 4 and 6 respectively. The epitopes were considered conserved in another genotype if it shows 100% identity across the epitope in at least 70% of sequences in that genotype in the randomly selected sequences used in this study, downloaded from the public database. In addition, epitope variants that were conserved in at least 70% of the sequences were analysed for conservancy for anchor residues at positions 2 and 9 for HLA class I and positions 1, 4, 6 and 9 for HLA class II.

### Validation of predicted epitopes

All the predicted epitopes were submitted to IEDB database (http://www.immuneepitope.org/) to confirm if they had been tested previously by other studies. The immuneepitope database contains experimentally confirmed data about antibody, T-cell epitopes, HLA binding, HLA restriction and HLA class.

### Common South African HLA alleles

For epitope prediction binding to common HLA alleles found in South Africa, the IEDB epitope analysis tool (http://tools.immuneepitope.org/tools/conservancy/iedb_input) was used for HLA class I using the artificial neural network (ANN) algorithm [66] on the IEDB server, while for Class II ProPred (http://www.imtech.res.in/raghava/propred/) at a 3% default threshold was used. ProPred predicts antigenic epitopes for 51 HLA class II alleles [54]. The most common South African alleles were found in published literature [67].

## Abbreviations

HCV: Hepatitis C virus
HLA: human leukocyte antigen.

## References

[1] World health Organization [WHO] (2014). Guidelines for the screening care and treatment of persons with hepatitis C infection.

[2] Pawlotsky JM (2003). The nature of interferon-alpha resistance in hepatitis C virus infection. Curr Opin Infect Dis.

[3] Ip PP, Nijman HW, Wilschut J, Daemen T (2012). Therapeutic vaccination against chronic hepatitis C virus infection. Antiviral Res.

[4] Ashfaq UA, Javed T, Rehman S, Nawaz Z, Riazuddin S (2011). An overview of HCV molecular biology, replication and immune responses. Virol J.

[5] Singh R, Kaul R, Kaul A, Khan K (2007). A comparative review of HLA associations with hepatitis B and C viral infections across global populations. World J Gastroenterol.

[6] Thio CL, Nolt KR, Astemborski J, Vlahov D, Nelson KE, Thomas DL (2000). Screening for hepatitis C virus in human immunodeficiency virus-infected individuals. J Clin Microbiol.

[7] Fanning LJ, Kenny-Walsh E, Shanahan F (2004). Persistence of hepatitis C virus in a white population: associations with human leukocyte antigen class 1. Hum Immunol.

[8] McKiernan SM, Hagan R, Curry M, McDonald GS, Kelly A, Nolan N, Walsh A, Hegarty J, Lawlor E, Kelleher D (2004). Distinct MHC class I and II alleles are associated with hepatitis C viral clearance, originating from a single source. Hepatology.

[9] Neumann-Haefelin C, McKiernan S, Ward S, Viazov S, Spangenberg HC, Killinger T, Baumert TF, Nazarova N, Sheridan I, Pybus O, von Weizsäcker F, Roggendorf M, Kelleher D, Klenerman P, Blum HE, Thimme R (2006). Dominant influence of an HLA-B27 restricted CD8+ T cell response in mediating HCV clearance and evolution. Hepatology.

[10] Hong X, Yu RB, Sun NX, Wang B, Xu YC, Wu GL (2005). Human leukocyte antigen class II DQB1*0301, DRB1*1101 alleles and spontaneous clearance of hepatitis C virus infection: a meta-analysis. World J Gastroenterol.

[11] Lechner F, Wong DK, Dunbar PR, Chapman R, Chung RT, Dohrenwend P, Robbins G, Phillips R, Klenerman P, Walker BD (2000). Analysis of successful immune responses in persons infected with hepatitis C virus. J Exp Med.

[12] Spada E, Mele A, Berton A, Ruggeri L, Ferrigno L, Garbuglia AR, Perrone MP, Girelli G, Del Porto P, Piccolella E, Mondelli MU, Amoroso P, Cortese R, Nicosia A, Vitelli A, Folgori A (2004). Multispecific T cell response and negative HCV RNA tests during acute HCV infection are early prognostic factors of spontaneous clearance. Gut.

[13] Lavillette D, Morice Y, Germanidis G, Donot P, Soulier A, Pagkalos E, Sakellariou G, Intrator L, Bartosch B, Pawlotsky JM, Cosset FL (2005). Human serum facilitates hepatitis C virus infection, and neutralizing responses inversely correlate with viral replication kinetics at the acute phase of hepatitis C virus infection. J Virol.

[14] Petrovic D, Dempsey E, Doherty DG, Kelleher D, Long A (2012). Hepatitis C virus-T-cell responses and viral escape mutations. Eur J Immunol.

[15] Shoukry NH, Grakoui A, Houghton M, Chien DY, Ghrayeb J, Reimann KA, Walker CM (2003). Memory CD8+ T cells are required for protection from persistent hepatitis C virus infection. J Exp Med.

[16] Klenerman P, Thimme R (2012). T cell responses in hepatitis C: the good, the bad and the unconventional. Gut.

[17] Brown RJ, Tarr AW, McClure CP, Juttla VS, Tagiuri N, Irving WL, Ball JK (2007). Cross-genotype characterization of genetic diversity and molecular adaptation in hepatitis C virus envelope glycoprotein genes. J Gen Virol.

[18] Patronov A, Doytchinova I (2013). T-cell epitope vaccine design by immunoinformatics. Open Biol.

[19] Dimitrov I, Flower DR, Doytchinova I (2011). Improving in Silico Prediction of Epitope Vaccine Candidates by Union and Intersection of Single Predictors. W J V.

[20] Gededzha MP, Selabe SG, Kyaw T, Rakgole NJ, Blackard JT, Mphahlele MJ (2012). Introduction of new subtypes and variants of hepatitis C virus genotype 4 in South Africa. J Med Virol.

[21] Antaki N, Craxi A, Kamal S, Moucari R, Van der Merwe S, Haffar S, Gadano A, Zein N, Lai CL, Pawlotsky JM, Heathcote EJ, Dusheiko G, Marcellin P (2010). The neglected hepatitis C virus genotypes 4, 5 and 6: an international consensus report. Liver Int.

[22] Bukh J, Apgar CL, Engle R, Govindarajan S, Hegerich PA, Tellier R, Wong DC, Elkins R, Kew MC (1998). Experimental infection of chimpanzees with hepatitis C virus of genotype 5a: genetic analysis of the virus and generation of a standardized challenge pool. J Infect Dis.

[23] Chamberlain RW, Adams NJ, Taylor LA, Simmonds P, Elliott RM (1997). The complete coding sequence of hepatitis C virus genotype 5a, the predominant genotype in South Africa. Biochem Biophys Res Commun.

[24] Gededzha MP, Selabe SG, Blackard JT, Kyaw T, Mphahlele MJ (2014). Near full-length genome analysis of HCV genotype 5 strains from South Africa. Infect Genet Evol.

[25] Himoudi N, Abraham J, Fournillier A, Lone YC, Joubert A, Op De Beeck A, Freida D, Lemonnier F, Kieny MP, Inchauspé G (2002). Comparative vaccine studies in HLA-A2.1-transgenic mice reveal a clustered organization of epitopes presented in hepatitis C virus natural infection. J Virol.

[26] Ohno S, Moriya O, Yoshimoto T, Hayashi H, Akatsuka T, Matsui M (2006). Immunogenic variation between multiple HLA-A*0201-restricted, Hepatitis C Virus-derived epitopes for cytotoxic T lymphocytes. Viral Immunol.

[27] Engler OB, Schwendener RA, Dai WJ, Wölk B, Pichler W, Moradpour D, Brunner T, Cerny A (2004). A liposomal peptide vaccine inducing CD8+ T cells in HLA-A2.1 transgenic mice, which recognise human cells encoding hepatitis C virus (HCV) proteins. Vaccine.

[28] Ibe M, Sakaguchi T, Tanaka K, Saito S, Yokota S, Tanaka T, Shimotohno K, Chujoh Y, Shiratori Y, Omata M, Miwa K, Takiguchi M (1998). Identification and characterization of a cytotoxic T cell epitope of hepatitis C virus presented by HLA-B*3501 in acute hepatitis. J Gen Virol.

[29] Giugliano S, Oezkan F, Bedrejowski M, Kudla M, Reiser M, Viazov S, Scherbaum N, Roggendorf M, Timm J (2009). Degree of cross-genotype reactivity of hepatitis C virus-specific CD8+ T cells directed against NS3. Hepatology.

[30] Park S, Shin E, Capone S, Caggiari L, Re VD, Nicosia A, Folgori A, Rehermann B (2012). Successful vaccination induces multifunctional memory T-cell precursors associated with early control of hepatitis C virus. Gastroenterology.

[31] Boucherma R, Kridane-Miledi H, Bouziat R, Rasmussen M, Gatard T, Langa-Vives F, Lemercier B, Lim A, Bérard M, Benmohamed L, Buus S, Rooke R, Lemonnier FA (2013). HLA-A*01:03, HLA-A*24:02, HLA-B*08:01, HLA-B*27:05, HLA-B*35:01, HLA-B*44:02, and HLA-C*07:01 monochain transgenic/H-2 class I null mice: novel versatile preclinical models of human T cell responses. J Immunol.

[32] Doi H, Hiroishi K, Shimazaki T, Eguchi J, Baba T, Ito T, Matsumura T, Nozawa H, Morikawa K, Ishii S, Hiraide A, Sakaki M, Imawari M (2009). Magnitude of CD8 T-cell responses against hepatitis C virus and severity of hepatitis do not necessarily determine outcomes in acute hepatitis C virus infection. Hepatol Res.

[33] Wentworth PA, Sette A, Celis E, Sidney J, Southwood S, Crimi C, Stitely S, Keogh E, Wong NC, Livingston B, Alazard D, Vitiello A, Grey HM, Chisari FV, Chesnut RW, Fikes J (1996). Identification of A2-restricted hepatitis C virus-specific cytotoxic T lymphocyte epitopes from conserved regions of the viral genome. Int Immunol.

[34] Wang S, Buchli R, Schiller J, Gao J, VanGundy RS, Hildebrand WH, Eckels DD (2010). Natural epitope variants of the hepatitis C virus impair cytotoxic T lymphocyte activity. World J Gastroenterol.

[35] Fitzmaurice K, Petrovic D, Ramamurthy N, Simmons R, Merani S, Gaudieri S, Sims S, Dempsey E, Freitas E, Lea S, McKiernan S, Norris S, Long A, Kelleher D, Klenerman P (2011). Molecular footprints reveal the impact of the protective HLA-A*03 allele in hepatitis C virus infection. Gut.

[36] Scognamiglio P, Accapezzato D, Casciaro MA, Cacciani A, Artini M, Bruno G, Chircu ML, Sidney J, Southwood S, Abrignani S, Sette A, Barnaba V (1999). Presence of effector CD8+ T cells in hepatitis C virus-exposed healthy seronegative donors. J Immunol.

[37] Chang KM, Gruener NH, Southwood S, Sidney J, Pape GR, Chisari FV, Sette A (1999). Identification of HLA-A3 and -B7-restricted CTL response to hepatitis C virus in patients with acute and chronic hepatitis C. J Immunol.

[38] Sreenarasimhaiah J, Jaramillo A, Crippin J, Lisker-Melman M, Chapman WC, Mohanakumar T (2003). Concomitant augmentation of type 1 CD4+ and CD8+ T-cell responses during successful interferon-alpha and ribavirin treatment for chronic hepatitis C virus infection. Hum Immunol.

[39] Matsui M, Moriya O, Belladonna ML, Kamiya S, Lemonnier FA, Yoshimoto T, Akatsuka T (2004). Adjuvant activities of novel cytokines, interleukin-23 (IL-23) and IL-27, for induction of hepatitis C virus-specific cytotoxic T lymphocytes in HLA-A*0201 transgenic mice. J Virol.

[40] Mizukoshi E, Nascimbeni M, Blaustein JB, Mihalik K, Rice CM, Liang TJ, Feinstone SM, Rehermann B (2002). Molecular and immunological significance of chimpanzee major histocompatibility complex haplotypes for hepatitis C virus immune response and vaccination studies. J Virol.

[41] Zhang J, Yamada O, Yoshida H, Iwai T, Araki H (2002). Autogenous translational inhibition of core protein: implication for switch from translation to RNA replication in hepatitis C virus. Virology.

[42] Matsueda S, Yamada A, Takao Y, Tamura M, Komatsu N, Yutani S, Ide T, Sata M, Itoh K (2007). A new epitope peptide derived from hepatitis C virus 1b possessing the capacity to induce cytotoxic T-lymphocytes in HCV1b-infected patients with HLA-A11, −A31, and -A33. Cancer Immunol Immunother.

[43] De Groot AS, Sbai H, Aubin CS, McMurry J, Martin W (2002). Immuno-informatics: Mining genomes for vaccine components. Immunol Cell Biol.

[44] Singh H, Raghava GP (2003). ProPred1: prediction of promiscuous MHC Class-I binding sites. Bioinformatics.

[45] Singh H, Raghava GP (2001). ProPred: prediction of HLA-DR binding sites. Bioinformatics.

[46] Toussaint NC, Dönnes P, Kohlbacher O (2008). A mathematical framework for the selection of an optimal set of peptides for epitope-based vaccines. PLoS Comput Biol.

[47] Zeng R, Li G, Ling S, Zhang H, Yao Z, Xiu B, He F, Huang R, Wei L (2009). A novel combined vaccine candidate containing epitopes of HCV NS3, core and E1 proteins induces multi-specific immune responses in BALB/c mice. Antiviral Res.

[48] Hu CT (2005). Vaccine Development for Hepatitis C: Lessons from the Past Turn into Promise for the Future. Tzu Chi Med J.

[49] Shehzadi A, Ur Rehman S, Idrees M (2011). Promiscuous prediction and conservancy analysis of CTL binding epitopes of HCV 3a viral proteome from Punjab Pakistan: an in silico approach. Virol J.

[50] Vallet S, Gouriou S, Nousbaum JB, Legrand-Quillien MC, Goudeau A, Picard B (2005). Genetic heterogeneity of the NS3 protease gene in hepatitis C virus genotype 1 from untreated infected patients. J Med Virol.

[51] Cento V, Mirabelli C, Salpini R, Dimonte S, Artese A, Costa G, Mercurio F, Svicher V, Parrotta L, Bertoli A, Ciotti M, Di Paolo D, Sarrecchia C, Andreoni M, Alcaro S, Angelico M, Perno CF, Ceccherini-Silberstein F (2012). HCV genotypes are differently prone to the development of resistance to linear and macrocyclic protease inhibitors. PLoS One.

[52] Torresi J, Johnson D, Wedemeyer H (2011). Progress in the development of preventive and therapeutic vaccines for hepatitis C virus. J Hepatol.

[53] Eckels DD, Bian T, Gill JC, Sønderstrup G (2002). Epitopes of the NS3 protein of hepatitis C virus: recognition in HLA-DR4 transgenic mice. Immunol Cell Biol.

[54] Day CL, Lauer GM, Robbins GK, McGovern B, Wurcel AG, Gandhi RT, Chung RT, Walker BD (2002). Broad specificity of virus-specific CD4+ T-helper-cell responses in resolved hepatitis C virus infection. J Virol.

[55] Hakamada T, Funatsuki K, Morita H, Ugajin T, Nakamura I, Ishiko H, Matsuzaki Y, Tanaka N, Imawari M (2004). Identification of novel hepatitis C virus-specific cytotoxic T lymphocyte epitopes by ELISpot assay using peptides with human leukocyte antigen-A*2402-binding motifs. J Gen Virol.

[56] Wertheimer AM, Miner C, Lewinsohn DM, Sasaki AW, Kaufman E, Rosen HR (2003). Novel CD4+ and CD8+ T-cell determinants within the NS3 protein in subjects with spontaneously resolved HCV infection. Hepatology.

[57] Vertuani S, Bazzaro M, Gualandi G, Micheletti F, Marastoni M, Fortini C, Canella A, Marino M, Tomatis R, Traniello S, Gavioli R (2002). Effect of interferon-alpha therapy on epitope-specific cytotoxic T lymphocyte responses in hepatitis C virus-infected individuals. Eur J Immunol.

[58] Smyk-Pearson S, Tester IA, Lezotte D, Sasaki AW, Lewinsohn DM, Rosen HR (2006). Differential antigenic hierarchy associated with spontaneous recovery from hepatitis C virus infection: implications for vaccine design. J Infect Dis.

[59] Hammer J, Belunis C, Bolin D, Papadopoulos J, Walsky R, Higelin J, Danho W, Sinigaglia F, Nagy ZA (1994). High-affinity binding of short peptides to major histocompatibility complex class II molecules by anchor combinations. Proc Natl Acad Sci U S A.

[60] Yusim K, Kesmir C, Gaschen B, Addo MM, Altfeld M, Brunak S, Chigaev A, Detours V, Korber BT (2002). Clustering patterns of cytotoxic T-lymphocyte epitopes in human immunodeficiency virus type 1 (HIV-1) proteins reveal imprints of immune evasion on HIV-1 global variation. J Virol.

[61] Vider-Shalit T, Raffaeli S, Louzoun Y (2007). Virus-epitope vaccine design: informatic matching the HLA-I polymorphism to the virus genome. Mol Immunol.

[62] Molero-Abraham M, Lafuente EM, Flower DR, Reche PA (2013). Selection of conserved epitopes from hepatitis C virus for pan-populational stimulation of T-cell responses. Clin Dev Immunol.

[63] Doytchinova IA, Guan P, Flower DR (2006). EpiJen: a server for multistep T cell epitope prediction. BMC Bioinformatics.

[64] Hertz T, Nolan D, James I, John M, Gaudieri S, Phillips E, Huang JC, Riadi G, Mallal S, Jojic N (2011). Mapping the landscape of host-pathogen coevolution: HLA class I binding and its relationship with evolutionary conservation in human and viral proteins. J Virol.

[65] Hall TA (1999). BioEdit: a user-friendly biological sequence alignment editor and analysis program for Windows 95/98/NT. Nucl Acids Symp Ser.

[66] Nielsen M, Lundegaard C, Worning P, Lauemoller SL, Lamberth K, Buus S, Brunak S, Lund O (2003). Reliable prediction of T-cell epitopes using neural networks with novel sequence representations. Protein Sci.

[67] Paximadis M, Mathebula TY, Gentle NL, Vardas E, Colvin M, Gray CM, Tiemessen CT, Puren A (2012). Human leukocyte antigen class I (A, B, C) and II (DRB1) diversity in the black and caucasian South African population. Hum Immunol.

